# Solitary Angiokeratoma in a Young Man: A Rare Case Report

**DOI:** 10.7759/cureus.37790

**Published:** 2023-04-18

**Authors:** Mohammad A Alghamdi

**Affiliations:** 1 Internal Medicine, Faculty of Medicine, Albaha University, Albaha, SAU

**Keywords:** angiokeratoma, cutaneous vascular disorder, plaque, circumscriptum, melanoma

## Abstract

Angiokeratoma is a rare vascular cutaneous disorder that usually presents as mostly asymptomatic aside from multiple dark red to blue or black papules over the skin in several clinically distinct conditions. Very rarely, it occurs in solitary localized forms that clinically mimic vascular disorders or sometimes melanoma. Solitary cutaneous angiokeratoma may result from damage to a venule's wall in the papillary dermis. This case study describes a 28-year-old male with a single angiokeratoma on the lateral aspect of his upper thigh and a clinical suspicion of a cutaneous melanocytic tumor. This case is intended to raise awareness about such rare skin lesions and the importance of histopathological examination.

## Introduction

Angiokeratoma (AK) is a benign tumor composed of dermal ectatic capillaries with overlying acanthotic and hyperkeratotic epidermal tissue. It can appear as a variety of vascular keratotic black lesions that differ in size, depth, and site [1]. There are five widely described types of AKs. Solitary or multiple AKs commonly occur on lower limbs, while Fordyce AK occurs on the vulva or scrotum. AK corporis diffusum is typically distributed in the lower trunk area, Mibelli AK occurs on the lateral and dorsal sides of the fingers and toes, and AK circumscriptum usually occurs unilaterally on the trunk, arms, or legs [2]. AK corporis diffusum is a condition that is linked to a lack of enzymes involved in metabolism and is known to occur in different illnesses [3]. The first variant, solitary AK, is rare.

Clinically, solitary AKs sometimes mimic angiomas or hematomas and show no distinguishing clinical features in comparison with these frequent skin lesions. However, some cases can raise suspicion for melanoma or dysplastic nevus, especially when the vessels are thrombosed. Thus, the way to reach an accurate diagnosis is a histopathological analysis based on the classical changes occurring in the epidermis overlying the ecstatic vascular channels, this ectasia in the papillary dermis is considered the main pathogenesis of this disorder [4,5]. We report a rare case of solitary AK in the present study.

## Case presentation

A 28-year-old male presented with a reddish-to-blackish elevated lesion that started on the upper lateral part of the right thigh and had been increasing in size for a year. The lesion was slowly progressing, lately painful, and reached more than 1 cm in size with an erythematous halo. The patient had no history of similar lesions and no history of trauma or injury to the affected site.

**Figure 1:**
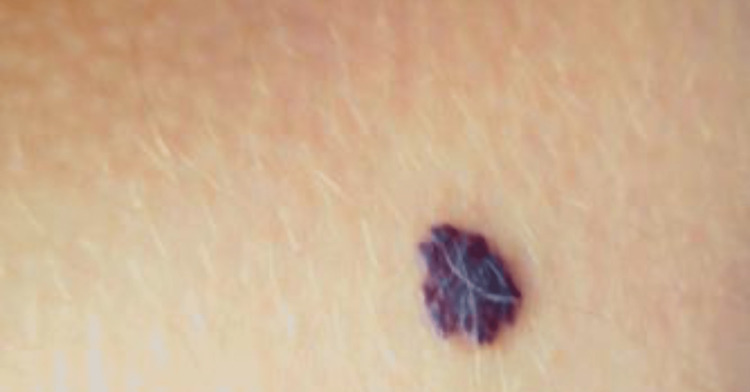
A well-defined reddish-to-blackish plaque on the upper part of the right thigh

Clinical examination revealed a solitary well-defined red-black plaque with a maximal diameter of 12 mm and no tenderness or ulceration. The lesion was firm in consistency and showed a halo with erythematous skin on the periphery (Figure 1). No abnormalities could be seen in the inguinal lymph nodes.

Clinical differential diagnoses included pseudolymphomatous AK, targetoid hemosiderotic hemangioma, and cutaneous melanocytic tumor. AKs that are thrombosed or dark in color could be mistaken for cutaneous melanoma. 

**Figure 2:**
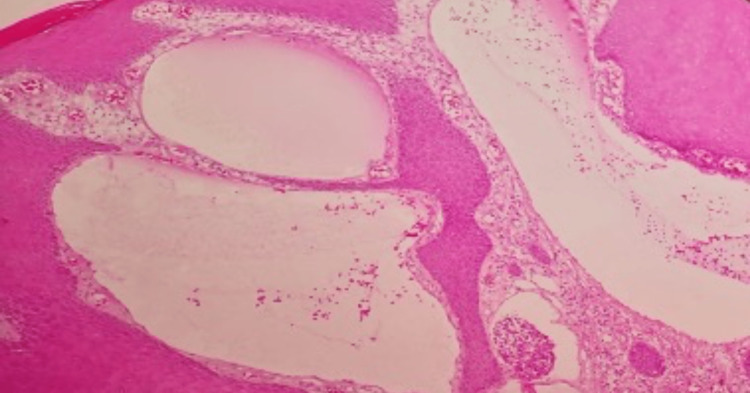
A histopathology picture showing hyperkeratosis, acanthosis, elongation of the rete ridges of the epidermis, and dilated dermal vascular spaces with no atypia (H&E, 100x)

The lesion was excised under local anesthesia, fixed for 24 hours in buffered formaldehyde, and prepared for paraffin-embedded processing. Serial sections were stained using ordinary hematoxylin-eosin stains and then examined under a light microscope. The examination revealed hyperkeratosis, acanthosis, and papillomatosis of the overlying epidermis with underlying dilated vascular spaces within the papillary dermis (Figure 2). 

In some of the vascular lumens, we found small thrombi along with scattered inflammatory cells. The main differential diagnosis raised on histological grounds was lymphangioma. Upon consultation with a histopathologist, a final diagnosis of solitary AK was determined. The patient’s clinical, radiological, and laboratory investigations were unremarkable. The patient was discharged, and at the next follow-up visit at six months, he showed no significant recurrence of any related disorders. 

## Discussion

AKs are seemingly benign vascular lesions characterized by dilated superficial vascular structures (blood vessels) underlying hyperkeratosis. With the exception of AK circumscriptum, which results from malformation of dermal capillaries, AKs are a consequence of ectatic dilation of pre-existing capillaries in the papillary dermis [1]. The first case was diagnosed in 1896, by John Addison Fordyce, however, the first classification of AK was developed in February 1967. The condition has had a global occurrence rate of 0.16% with a slightly higher occurrence in males [6,7]. 

AKs of the scrotum and vulva (AK of Fordyce) are single or numerous lesions that range in color from red-purple to black and develop along superficial vessels of the scrotum and vulva. Scrotal lesions may also be related to inguinal hernias, varicoceles, and thrombophlebitis. Oral contraceptive use, vulvar varicosities, hemorrhoids, and elevated venous pressure during pregnancy can all be linked to vulvar lesions. AK corporis diffusum is characterized by a few to numerous, frequently grouped AKs that usually affect the lower trunk area (lower abdomen, hips, thighs, and genitals). They are known to occur in healthy people as well as those with various enzyme-deficiency illnesses, such as Fabry disease. 

AK of Mibelli is composed of multiple hyperkeratotic papules and plaque, which are most frequently found on the lateral and dorsal sides of the fingers and toes. They can also affect the hands and feet. Chilblains and acrocyanosis could be associated with AK of Mibelli. AK circumscriptum is characterized by numerous distinct papules or hyperkeratotic papules and nodules that are frequently confluent. They are most often unilateral and affect the trunk, arms, or legs. Lastly, solitary or multiple AKs are single or multiple well-defined plaques that are red-black in color and commonly found on the lower limbs. Solitary lesions can be mistaken for melanoma [8-11]. Although they differ clinically in their appearance, morphology, and anatomical localization, solitary AK lesions and melanoma share similar histological features. Blood vessel thrombosis is common and is the first feature responsible for it being mistaken for melanocytic tumors, including malignant melanoma [12]. 

Histological diagnosis is an important step in diagnosing and detecting unusual skin lesions all over the body, including the upper thigh and perianal area, which may be neglected or missed [13,14]. Histologically, cutaneous AK demonstrates hyperkeratosis and acanthosis of the overlying epithelium with papillary vascular dilation. The only difference between the histological pictures of cutaneous and oral AKs is that hyperparakeratosis is evident in oral lesions, whereas hyperorthokeratosis can be observed in skin lesions [15]. Increased proliferative activity normally takes place on the surface of vascular malformations that are adjacent to the epidermis or epithelium, and it has been believed to cause this reactive epidermal or oral epithelium development [11]. On contrary, lymphangioma is formed of dilated lymphatic spaces lined by a single attenuated layer composed of endothelial cells involving the dermis, subcutis, and sometimes the underlying fascia and skeletal muscle.

Solitary AKs and targetoid hemosiderotic hemangiomas (THHs) are acquired vascular malformations caused by superficial vascular dilatation that may result from trauma. On the lower limbs, they both frequently develop a brownish or blackish papule that looks like a melanocytic lesion. Both have extravasated red blood cells, hemosiderin deposits, ectatic papillary dermal vessels, lymphocytic infiltrates, and lymphangiectasias with overlaying hyperplasia. THHs are larger, have more pronounced dermal vessel alterations, often have dissected vascular empty spaces, and have greater hemosiderin deposits than solitary AKs [8].

Surgical excision is the most common and effective treatment for AKs. The two goals of this procedure are simultaneously removing the tumor and confirming the diagnosis [16]. Other treatment options are diathermy, intralesional steroid injection, radiation, and laser ablations [5, 17], which can be used together or independently.

## Conclusions

We have presented an uncommon case of a solitary cutaneous AK in the upper lateral part of the thigh. The lesion was clinically suspected to be a melanocytic tumor but was histologically verified to be AK without any other accompaniment pathological lesions. The lesion was effectively excised under local anesthesia, and there was no clinical recurrence. Histopathology examination is crucial for any excised skin lesion.
